# Physicochemical design of nanobiomaterials in oncology: structure-to-function principles for sequential transport and delivery

**DOI:** 10.3389/fonc.2026.1817437

**Published:** 2026-04-29

**Authors:** Athina Angelopoulou, Mauro Ferrari, Qingxin Mu

**Affiliations:** 1 Laboratory of Pharmaceutical Technology, Department of Pharmacy, University of Patras, Patras, Greece; 2 Laboratory of Metabolic Engineering and Systems Biology, Institute of Chemical Engineering Sciences, Foundation for Research and Technology-Hellas (FORTH/ICE-HT), Patras, Greece; 3 BrYet US, Inc., Houston, TX, United States; 4 Department of Pharmaceutics, University of Washington, Seattle, WA, United States

**Keywords:** biological effects, nanobiomaterials, nanomedicine, phenotype targeting, physicochemical properties, structure-function relationships, tumor microenvironment (TME)

## Abstract

The physicochemical properties of nanobiomaterials (NBM) have enabled nanomedicine platforms capable of addressing major therapeutic challenges in oncology, including poor bioavailability, systemic toxicity, limited tumor accumulation, and off-target effects. Through coordinated optimization of size, shape, and surface chemistry, NBM support controlled drug release, improved targeting strategies, and modulation of the tumor microenvironment (TME). This structure-to-function tuning further governs their interaction with biological transport barriers, enabling navigation across abnormal vasculature, dense extracellular matrix, and elevated interstitial fluid pressure. This review critically examines key physicochemical properties (size, shape, and surface chemistry) that govern nano-bio interactions and influence circulation, cellular uptake, biodistribution, immune evasion, and intracellular transport in solid tumors. Emphasis is placed on structure-to-function relationships that link material design with therapeutic performance. The integration of physicochemical optimization with the distinct transport phenotypes of TME highlights that effective NBM require coordinated tuning of multiple parameters rather than merely design adjustments. The synergistic modulation of physicochemical properties can produce adaptive, multifunctional NBM capable of engaging specific TME components and overcoming critical barriers. Together, these insights establish the foundation for rational NBM engineering and provide the mechanistic basis for tumor phenotype focused strategies.

## 1 Introduction

The progress in cancer nanotechnology effectively transformed nanobiomaterials (NBM) from passive carriers to multifunctional, adaptive nanomedicines capable of promoting dynamic therapeutic responses in the complex tumor microenvironment (TME). Through their innovative design, NBM can support cellular and molecular biological interactions at the cell-cell and cell-material interface within TME, which are the main determinants of therapeutic outcome (1). The growing interest in NBM stems from their prolonged blood circulation, biocompatibility and ease of functionalization which allow modulation of therapeutic responses, overcoming the limitations of systemic toxicity and off-target effects. Thus, NBMs offer the opportunity to reshape traditional chemotherapeutic agents (1, 2). The increasing research progress in NBM, has enabled the design of multifunctional platforms capable of integrating theranostic functions that respond to physiological stimuli, such as pH, enzymes, redox, and evade immune clearance through biomimetic approaches. The biomimetic drug delivery design has expanded into inorganic particles such as gold and iron oxide nanostructures, soft materials such as hydrogels and dendrimers, and bio-inspired systems such as exosomes and membrane-coated particles that enable precise control over spatial and temporal drug release and synergistic combination therapies. Such modern NBM are engineered to interact with the TME to address major challenges within the biological milieu including, tumor penetration, multidrug resistance (MDR) and immunosuppression, while maintaining favorable biocompatibility and minimizing systemic toxicity (2, 3). Specifically, the barriers encountered by the heterogeneous TME of solid tumors, such as abnormal vasculature, high interstitial pressure, and dense extracellular matrix (ECM) may create transport-limiting conditions that restrict the penetration and efficacy of NBM, highly promoting poor availability and MDR (4). The amenability of NBM to be easily tuned for their physicochemical properties, such as size, shape, surface charge, functionalization, and drug delivery capacity, has significantly improved their tumor targeting and selectivity, maximizing their ability to overcome the intrinsic TME obstacles (5).

Lately, a significant attempt has been made in research to broaden the understanding of how NBM interact with the complex biological milieu of TME (6, 7). The physicochemical parameters are no longer designed independently but in correlation with the therapeutic goals and the specific biological challenges presented by each tumor type (8, 9). This evolution reflects that integrated approaches which bridge the gap between materials science, pharmaceutical technology and systems’ biology are required for the next generation of NBM. In this review, the therapeutic efficacy of NBM will be discussed in relation to their structure and physicochemical profile. The interplay between NBM design and biological milieu, including blood circulation and biodistribution, cellular uptake, tumor penetration, and immune responses will be presented to frame a structure-to-function guide for cancer NBM development that addresses the solid clinical challenges of solid tumor. While fragile macromolecular cargos such as nucleic acids highlight certain delivery limitations, the structure-to-function framework described here equally applies to a wider set of therapeutic classes such as small molecules and peptide/protein drugs, radiotherapy-sensitizing nanocarriers, and transport phenotype-targeted platforms. These concepts together define realistic translational opportunities for NBM, since the structure-to-function framework is equally important for therapeutic classes that rely on physicochemical tuning to improve tumor accumulation and intracellular bioavailability. Recent advances in cancer phenotype targeting have also demonstrated that NBM can be engineered to exploit unique transport properties of tumor vasculature and stromal architecture, extending their application beyond genetic targets. These emerging approaches broaden the relevance of physicochemical design and reinforce the need for integrative strategies that account for the dynamic and heterogeneous nature of solid tumors (10).The physicochemical properties of NBM, including shape, surface charge, chemical composition and modification, can highly influence the biodistribution and pharmacokinetic profile of such nanomedicines and critically determine their biological and immunological profile (11–13). Main biological interactions of the NBM, such as the formation of protein corona (PC), the interplay with cellular uptake mechanisms, and endosomal escape efficiency can be optimized by engineering their physicochemical parameters (13–15). The PC, a dynamic layer of biomolecules adsorbed onto the NBM surface, serves as the primary biological interface determining their performance in drug delivery and cancer nanotherapy. As NPs meet the biological fluids, their surfaces are modified by protein binding, often leading to rapid recognition and clearance by the mononuclear phagocyte system (MPS). The composition and behavior of the PC are strongly influenced by the macroscopic physicochemical properties of the NBM (size, shape, surface chemistry and charge) that ultimately determine the dominant forces (electrostatic, hydrophobic, π-π stacking, hydrogen bonding, van der Waals) of the nano-bio interface. The dynamic PC between NBM and serum proteins is governed by time- and concentration- dependent kinetics that regulate adsorption and desorption mechanisms of biomacromolecules promoting a dynamic equilibrium exchange further affecting their pharmacokinetic and pharmacodynamic behavior (15, 16).

The size and shape of NBM are widely documented physicochemical properties that affect important biological pathways including cellular uptake, intracellular transportation, and tumor accumulation. Of the most important size-related biological barriers are the membrane bilayers (4–10 nm thickness), the nuclear pore complex (NPC, 80–120 nm diameter) and the size of endocytic vehicles, where phagocytosis and macro-pinocytosis internalize large particles or aggregates in contrast to receptor-mediated and non-specific endocytosis (17, 18). Despite significant advances in NBM research, many design strategies are still guided by the enhanced permeability and retention (EPR) effect, which describes the preferential accumulation of NBM within tumor tissues due to abnormal vascular permeability and impaired lymphatic drainage. Tumor vasculature fenestrations, which vary depending on tumor type and vascular maturity, permit the extravasation of NBM within an appropriate size range into the tumor interstitium. In general, NBM in the range of approximately 50–200 nm are often associated with favorable accumulation through EPR-driven transport in preclinical models, although the magnitude of this effect varies substantially across tumor types and experimental conditions (17–20). Importantly, the EPR effect alone does not guarantee efficient drug delivery, as successful extravasation must be followed by effective diffusion through the dense ECM and subsequent cellular internalization within the TME. In this context, NBM size presents a critical exchange between circulation, tumor accumulation, and tissue penetration (21, 22). Larger NBM may exhibit prolonged circulation and enhanced retention at tumor sites but are often limited in their ability to penetrate deeply into tumor tissue due to steric hindrance imposed by the dense ECM structure (23–26). In contrast, smaller nanoparticles generally demonstrate improved diffusion through the narrow interstitial spaces of the ECM, thereby supporting deeper tumor penetration, although excessively small particles may undergo rapid renal clearance, limiting their effective accumulation at the tumor site (27–31). These observations highlight that NBM size should not be considered a universal determinant of delivery performance, but rather a context-dependent parameter that must be optimized in relation to tumor transport characteristics and NBM physicochemical properties.

Furthermore, the particle shape and aspect ratio (AR, defined as the ratio of length to width or diameter) also affect transport mechanisms. Variations in AR have been reported to influence circulation time and cellular uptake behavior in several experimental systems. The shape and AR of NBM represent important geometric parameters in addition to size that can influence biodistribution, cellular uptake, and tumor penetration. For example, studies have shown that nanorods with higher AR may exhibit prolonged circulation times and enhanced internalization by cancer cells. However, very high AR values (e.g., above 4) have been reported to influence membrane wrapping kinetics during endocytosis, potentially affecting the internalization time of elongated NBM. Moreover, AR can influence tumor penetration ability of the NBM, as intermediate AR values (approximately 1 to 4) have been reported to facilitate surface ligand presentation and membrane wrapping dynamics. Such effects potentially support navigation of NBM through the ECM or adherence to endothelial surfaces (19, 20).

Alongside size and shape, the proper design of the surface properties (chemistry, functionalization, and surface charge) is essential for developing NBM that effectively exploit nano-bio interactions in the TME for effective cancer-targeted nanomedicines. Most biological processes relevant to NBM occur at the nano-bio interface, where the surface properties of NBM dictate their ability to navigate and interact with tumor components, including serum proteins, blood vessels, ECM components, macrophages, tumor associated macrophages (TAMs), cancer stem cells (CSCs), and with cellular components such as membrane phospholipids, endocytic vehicles, and organelles like mitochondria and nucleus (32). In the acidic and heterogeneous TME, surface modification of the NBM, such as coatings with biomimetic ligands, polymers, or proteins can improve target specificity, minimize immune clearance, and enhance tumor accumulation. In principle, nano-bio interactions should promote therapeutic delivery and minimize adverse effects by aligning nanoparticle design with the biophysical and biochemical properties of the target TME. Especially, in the TME a favorable electrostatic environment is created due to the negative membrane potential of cancer cell membranes that can highly increase the affinity and internalization of positively charged NBM via specific and non-specific endocytic routes. The increased secretion levels of lactate ions and sialic acid increase the negative membrane potential of cancer cells, facilitating the strong attraction of NBM with positive surface charge (33, 34). Moreover, hydrophobic-hydrophilic balance can influence the transportation mechanisms of NBM through the cellular membranes, especially within the TME where dense stromal ECM and abnormal vasculature are main obstacles. Tumor ECM structures a complex architecture presenting a physical and electrostatic barrier to NBM penetration due to the dense collagen-fibril network with inter space of 20–40 nm. Moreover, the basement membrane of ECM is a physical filter of surface charge due to its negatively charged components, such as chondroitin sulfate. As a result, the passage of NBM is restricted by size and charge with small and positively charged NBM being more likely to transport through tumor ECM. The surface functionalization of NBM with ligand-directed targeting (e.g., integrin-binding) and stealth coatings (e.g., PEG, albumin) has been proved strategic for evading dense ECM and facilitating deep tumor penetration (35, 36). Surface functionalization of NBM has played a key role in the induction of stimuli-responsiveness to tumor specific (pH, redox gradients, enzymes), or external stimulus (magnetic, PDT, PTT) (37).

Despite significant advances in NBM research, efficient delivery of therapeutics to solid tumors remains challenging, mainly due to the complex and heterogeneous transport barriers present within the TME. These barriers include abnormal and heterogeneous vasculature permeability, elevated interstitial fluid pressure, dense ECM, and dynamic cellular interactions. Collectively these barriers regulate the transport of NBMs and therapeutic agents from systemic circulation to tumor cells. Consequently, NBM design strategies optimized for a single mechanism of accumulation often fail to achieve effective intratumoral distribution and therapeutic delivery. This review addresses this challenge by organizing current knowledge according to the sequential transport barriers that govern NBM behavior in tumors. By integrating physicochemical design parameters such as size, shape, and surface chemistry with biological transport processes, we present a structure-to-function framework that aligns NBM properties with tumor transport phenotypes. Through this perspective, we aim to highlight that the design of NBM can be tailored to navigate complex tumor transport environments. Thus, ultimately supporting the development of next-generation nanomedicines with improved therapeutic performance and translational potential.

## 2 Key physicochemical parameters

Recent advances in NBM have shifted cancer therapy from non-specific systemic chemotherapy toward site-directed, multifunctional platforms designed to interface with complex TME (38). The key physicochemical parameters like size, shape, and surface chemistry influence critical biological processes including circulation, barrier traversal, cellular uptake and tissue penetration. These attributes remain central to rational NBM engineering across therapeutic modalities such as small molecules, peptide/protein drugs and radiotherapy-sensitizing agents. This design philosophy aligns directly with the cancer phenotype targeting concept, which focuses on dynamic functional traits such as abnormal transport, stromal remodeling, immune evasion, and metabolic reprogramming rather than static gene mutations (10). By exploiting transport and mechanical phenotypes across heterogeneous tumors and metastatic lesions, NBM can achieve more consistent therapeutic responses to support various therapeutic modalities. Despite the significant advances in NBM design, the clinical translation of NBM-based therapeutics has often produced mixed outcomes. Several clinically approved therapeutics have demonstrated improved pharmacokinetics and reduced systemic toxicity but only modest improvements in therapeutic efficacy compared with conventional formulations (39). Such results highlighted the complexity of tumor transport barriers within solid tumors. Heterogeneous tumor vasculature, elevated interstitial fluid pressure, and dense extracellular matrices frequently limit uniform drug distribution regardless of efficient systemic delivery. Thus, increasing attention has been directed toward design strategies that address these transport barriers in a sequential manner. As a result, multistage delivery and sequential transport phenotype-guided NBM approaches have been developed to address the barriers encountered between systemic circulation and intracellular drug release. Such considerations underscore the development of conceptual frameworks that integrate NBM physicochemical design with the physical and biological transport barriers present in tumors.

**Figure 1:**
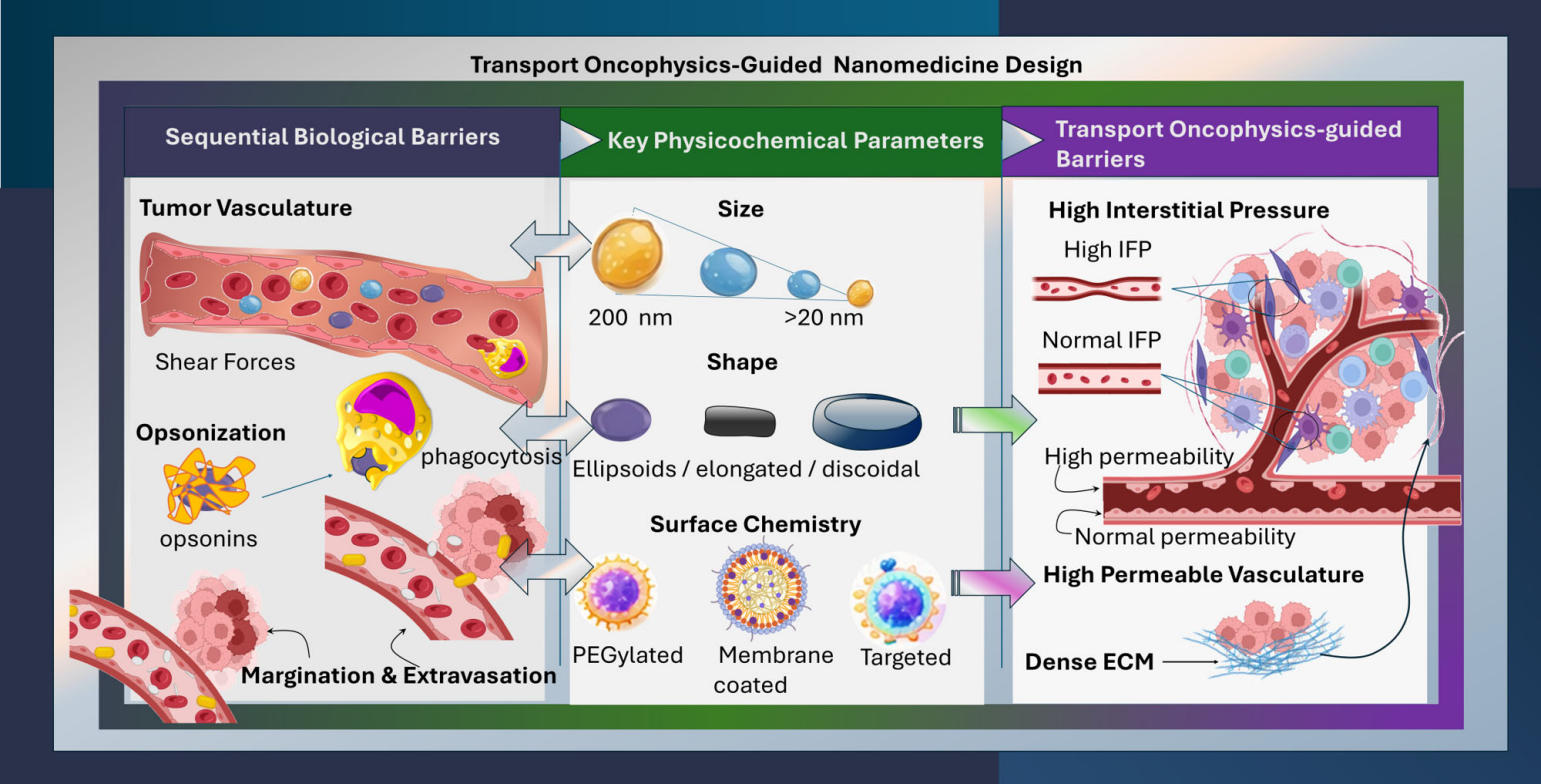
Transport Phenotype-guided design of NBM presenting the Structure-to-Function relationship. Sequential biological barriers, such as tumor vasculature, opsonization, and margination and extravasation guide rational tuning of NBM physicochemical properties to optimize delivery, based on dominant tumor transport barriers.

The biological barriers of TME, including abnormal vasculature, dense ECM, hypoxia, and high interstitial fluid pressure limit the performance of systemically administered therapeutics. Multifunctional NBM address these limitations by improving stability, enhancing the retention at tumor sites, and increasing the effective local drugs’ concentration such as small molecules, proteins, peptides and sensitizers (38). Thus, the incorporation of targeting ligands or cell-penetrating peptides can further facilitate cytosolic delivery of small molecules and proteins, while promoting the localized effect of radiosensitizers to hypoxic regions improving radiotherapy efficacy. For example, the combination of the small molecule canagliflozin with magnetic nanoparticles for the targeting of hypoxic tumors was reported to demonstrate strong therapeutic potential by acting as a radiosensitizer combined with radiotherapy to overcome the inherent resistance of low-oxygen solid tumors (40). Such structure-to-function interplays provide the foundation for designing NBM that selectively exploit tumor-associated phenotypes (38). Manipulating structure-to-function design is essential to overcoming the multifaceted TME challenges and advancing NBM therapeutic potential. A translational example is ML-016 (BrYet US, Inc.), a multi-component mesoporous silicon-based product currently entering Phase 1/2 clinical trials (41). ML-016 is based on an injectable nanoparticle generator-polymeric doxorubicin (iNPG-pDox) platform (42). In contrast to classical delivery systems, the iNPG-pDox functions as a sequential transport system, where discoidal porous silicon microparticles accumulate in the tumor through favorable hemodynamic margination and subsequently release a polymeric doxorubicin conjugate (pDox). These polymer conjugates self-assemble into nanoparticles locally within the TME, enabling deeper tissue penetration and intracellular drug release. Such targeting systems exemplify the principles of Transport Oncophysics (TOP) (visualized in **Figure 1**), where therapeutic delivery is conceived as a sequence of transport phenomena that must be navigated between systemic circulation and intracellular drug action. TOP lay emphasis on exploiting inherent variations in physical properties and transport phenotypes between healthy and pathological tissues to selectively deliver therapeutics (10, 43). Within this framework, NBM design focuses on aligning physicochemical properties with the dominant transport barriers that characterize different tumor phenotypes. Such NBMs are designed to encounter successive transport barriers instead of a single biological interaction.

The spatial tumor heterogeneity and restricted perfusion of solid tumors hinder passive and active drug targeting and necessitate deep penetration through a dense stromal tissue or across physiological barriers like the blood-brain barrier (44). Thus, NBM size remains an important parameter influencing biodistribution and cellular uptake. Moreover, surface chemistry and stealth coatings modulate protein corona formation, opsonization and endosomal escape, while ligand presentation supports receptor-mediated uptake and tissue selectivity (45–47). Representative formulations such as ligand-functionalized PLGA particles and targeted PEGylated liposomes demonstrate that coordinated tuning of size, and surface properties can improve delivery of chemotherapeutics, peptides and radiosensitizers across challenging TMEs (46, 48). The interplay of these parameters (summarized in **Table 1**) involves necessary compromises for optimal balance between physicochemical design and circulation and penetration. For example, maximizing circulation through size effects may reduce penetration, and enhancing uptake by surface chemistry may increase off-target clearance. Thus, rational design is required through multi-parameter evaluation supported by standardized assays and physiologically relevant models, such as organotypic ECM, tumor explants, immunocompetent animals, microfluidic chips. Ultimately, long-term safety and biodistribution studies through systematic *in vivo* evaluation are critical for translating these design strategies to progress. Finally, manufacturing reproducibility, quality control (e.g., CMC), and regulatory alignment require parallel progress for producing safe and scalable clinically effective nanomedicines. These physicochemical principles form the foundation for the structure-to-function strategies discussed in the following sections, where the interplay of physicochemical properties and biological interactions dictate differentiated NBM design. The physicochemical properties of NBM, including size, geometry, and surface composition, operate as interdependent variables that shape nanoparticle transport through the tumor microenvironment. Rather than acting independently, these parameters influence multiple biological processes, including circulation, vascular transportation, stromal penetration, and cellular internalization. The TOP framework highlights the importance of integrating these design parameters to align NBM properties with the sequential transport barriers encountered in tumor tissues.

**Table 1:** Structure-to-function physicochemical design strategies guiding phenotype-targeted NBM development.

Strategy category	Key physicochemical feature	Functional transport role	Representative NBM examples	Design rules/constraints	Refs
Size Optimization	Small size (<200 nm), ultrasmall (<20 nm)	Controls circulation time, endothelial transcytosis, and interstitial diffusion	Polymeric NPs, liposomes, micelles	Smaller size improves penetration but increases renal clearance	(27–31, 44)
Shape Engineering	Non-spherical (discoidal, platelet-like)	Enhances vascular margination and endothelial adhesion under flow	iNPG as silicon carriers for ML-016	Improved margination may reduce deep tissue diffusion	(10, 41–43)
Surface Chemistry (Stealth)	PEGylation, hydrophilic coatings	Reduces opsonization, prolongs circulation, stabilizes payload	PEGylated liposomes, PLGA-PEG	Excessive stealth may limit endothelial adhesion and uptake	(38, 45–48)
Ligand Functionalization	Peptides, targeting ligands, CPPs	Enables receptor-mediated uptake and tissue selectivity	Ligand-functionalized PLGA, liposomes	Ligand density may increase immune recognition or off-target binding	(45–47)
Multifunctional Integration	Injectable nanoparticle generator	Sequential transport across vascular, stromal, and cellular barriers	iNPG-pDox (ML-016)	Increased complexity complicates manufacturing and CMC	(41–43)
Payload-Carrier Coupling	Prodrugs, radiosensitizers	Enables localized activation and phenotype-specific therapy	pDox conjugates, hypoxia radiosensitizers	Requires precise release control and safety validation	(40–42)
Transport Oncophysics Framework	Coordinated tuning of size, shape, surface, mechanics	Exploits conserved transport phenotypes across tumors	Multistage silicon systems	Requires multi-parameter optimization and predictive models	(10, 43)

### 2.1 Size and shape as determinants of tumor accumulation and cellular uptake

Efficient delivery of NBM to solid tumors depends strongly on size and shape, which govern transport through blood flow, interaction with tumor vasculature, and intracellular entry. The abnormal and heterogeneous permeability of tumor vessels typically favors the passage of NBM of average size around 10–200 nm, although the optimal size for penetration may differ from the one for retention (48–50). Clearance pathways, including hepatic (liver) and renal (kidney), further determine NBM design since phagocytic cells in the liver and spleen and glomerular filtration in the kidneys support NBM breakdown and expel. Particles with a size of < 8 nm undergo renal elimination, while larger or rigid particles tend to accumulate in the liver and spleen through macrophage-mediated uptake (51, 52). Thus, size determines not only tumor access but also systemic exposure and long-term safety. An additional design axis is added by shape anisotropy that influences margination and endothelial interactions. Elongated or disk-like geometries have high aspect ratios (length/width) thus experience higher lateral drift toward vessel walls due to increased hydrodynamic lift and torque, enhancing their probability of margination and binding to tumor endothelium (53, 54). Discoidal and ellipsoidal particles have been shown to exhibit markedly enhanced margination compared to spherical counterparts under laminar shear flow conditions, a behavior attributed to their rotational dynamics, inertial effects, and particle Stokes number (55, 56). These geometric principles formed the basis for the development of multistage vectors (MSVs), in which discoidal porous silicon microparticles are engineered to accumulate within tumor vasculature through margination effects. Subsequently, the MSVs release secondary nanoparticles such as liposomes or polymeric nanoparticles from their internal pores. At a third stage, the secondary nanoparticles release their payloads. This architecture enables staged drug delivery where the microparticle carrier first localizes to the tumor vasculature and then releases therapeutic nanoparticles that penetrate deeper into tumor tissue (57–59).

Geometry is also a determinant of cellular internalization affecting endocytic pathways like clathrin-mediated and macropinocytosis and intracellular transportation through the dense ECM. Specifically, NBMs with size < 40 nm navigate the dense ECM more effectively and penetrate deeper into tumor tissue due to reduced steric hindrance and improved diffusion (60, 61). NBM with sizes around 50–200 nm are commonly internalized through clathrin- (100–200 nm) and caveolae-mediated (50–100 nm) endocytic pathways. However, within this size range, penetration into poorly perfused tumor regions may remain limited due to restricted diffusion through the dense ECM (62, 63). Larger NBM (>500 nm) are often internalized through macropinocytosis, a process involving the non-selective engulfment of extracellular fluid and particles, a pathway independent of size-related limitations (64, 65). Moreover, shape can modulate uptake efficiency, as spherical NBM are frequently reported to be readily internalized, whereas rod-like NBM with AR around 3–4 have been observed to exhibit stronger membrane adhesion and enhanced internalization compared with spherical constructs (64, 66). For example, PEG-coated gold nanorods have been shown to achieve longer systemic circulation and higher tumor accumulation compared with PEGylated spherical gold nanoparticles in tumor-bearing mice, indicating that tuning anisotropic geometry can enhance biodistribution and tumor uptake *in vivo* (67). Moreover, *in vitro* studies comparing anisotropic gold nanoparticle geometries reported that shape strongly influences internalization pathways, with rods and other anisotropic shapes showing differential uptake characteristics compared with the spherical, suggesting geometry can be a modulator of endocytosis mechanisms (68).

Small-sized NBM (< 50 nm) have shown advantages in penetrating restrictive barriers such as the narrow interstitial spaces of extracellular matrix and the blood-brain barrier (BBB). In a compelling example, ligand-free PEGylated mesoporous silica nanoparticles (size around 25–50 nm) achieved superior BBB penetration and significantly increased brain tumor accumulation relative to free Doxorubicin. This study underscored the value of sub-50 nm engineered NBM for diffusion-limited environments promoting significantly improved tumor inhibition and survival in glioma models (69). The understanding of underlying mechanisms necessitates further studies for the contributions of paracellular diffusion and other brain transportation pathways in comparison to particles’ size. The comparison of 20, 40, and 80 nm MSNs functionalized with cRGD peptides for doxorubicin delivery revealed that ~40 nm particles achieved the most favorable balance between BBB penetration, tumor cell internalization, and retention, while limiting off-target effects to healthy brain tissue (70). Thus, particle size tuning is greatly important especially in the sub-50 nm regime, representing a strong design approach to overcome diffusion limitations and improve therapeutic outcomes (71, 72). Ultrasmall nanoparticles (< 10 nm) offer another distinct example, combining deep tissue penetration with rapid renal clearance that minimizes long-term accumulation (73). Their size facilitates diffusion through the narrow interstitial spaces (20 to 40 nm) of the dense ECM allowing access to hypoxic, poorly vascularized tumor loci with limited lymphatic drainage (74). These advantages boost the therapeutic efficacy of ultrasmall nanomedicines. Doxorubicin-conjugated ultrasmall gold nanoparticles (2 nm) demonstrated efficient BBB permeation and selective accumulation in GBM organoids. Notably, the ultrasmall nanoparticles showed reduced penetration to healthy tissue organoid (75). However, their limited drug-loading capacity and ultrafast clearance impose design constraints posing challenges for effective drug delivery. The clinical translation of ultrasmall NBM depends on careful optimization of surface chemistry and stealth coating to balance deep penetration and circulation time (76). In a recent study, ultrasmall gold nanoparticles (~5 nm) were functionalized with cleavable, antifouling triblock peptide layers that combined a CPP TAT peptide with a GFLG peptide cleavable by cathepsin B in the TME and an antifouling peptide. This smart ultrasmall NBM achieved enhanced tumor uptake, radiosensitization, and safe renal elimination, illustrating how surface chemistry and ultrasmall size synergize to overcome TME barriers and non-specific distribution (77).

Building on the crucial role of size tuning, particle shape is an equally important parameter to dictate performance. Shape anisotropy can be harnessed to further improve margination and endothelial targeting, evade macrophage uptake, reduce opsonization, and enhance tumor localization, complementing size-based design strategies. Anisotropic particles such as elongated, discoidal, and ellipsoids show superior vascular interaction due to hydrodynamic margination and prolonged circulation effectively increasing extravasation probability compared to the spherical ones (55, 56). In a systematic study of MSNs with varying morphologies (spherical, rod-like, and hexagonal) the hexagonal particles exhibited prolonged circulation time, superior pharmacokinetics, and tumor accumulation with reduced macrophage uptake (78). Moreover, particle shape can influence cellular uptake profile. Studies have reported that long rod-shaped MSNs may be internalized more efficiently than short rods or spherical counterparts under certain experimental conditions (79). Rod−like NBM with intermediate aspect ratios (approximately 3-4) have been reported to exhibit enhanced internalization, potentially attributed to increased surface area and prolonged contact with the cell membrane, which may facilitate receptor engagement and membrane wrapping. In a comparative study of spherical and rod-shaped gold theranostic nanoparticles loaded with cisplatin and radiolabeled iodine-125, rod-shaped particles functionalized with αvβ3-targeting RGD peptides achieved greater early tumor uptake and deeper interstitial diffusion than spherical analogues (80). Collectively, these findings highlight NBM shape in synergy with size and surface chemistry, is a powerful design axis to overcome barriers related to circulation, biodistribution, and tumor infiltration. The key size- and shape-dependent transport regimes governing circulation, biodistribution, tumor penetration, and cellular uptake are summarized in **Table 2**.

**Table 2:** Size- and shape-dependent transport mechanisms governing tumor accumulation, penetration, and cellular uptake of NBM.

Design axis	Size/shape regime	Dominant transport/uptake mechanism	Functional outcome in tumors	Representative examples	Key limitations/rules	Refs
Size	< 8–10 nm (ultrasmall)	Renal filtration, rapid diffusion through ECM	Deep tissue penetration, fast systemic clearance	Ultrasmall AuNPs, peptide-coated AuNPs	Limited drug loading, ultrafast clearance	(51, 73–77)
	10–40 nm	Paracellular diffusion, efficient ECM navigation	Improved penetration in dense ECM and BBB	Sub-50 nm MSNs, PEGylated silica	Reduced retention in leaky tumors	(60, 61, 69–72)
	50–100 nm	Caveolae-mediated endocytosis, balanced PK	Efficient uptake with moderate penetration	PEGylated liposomes, PLGA NPs	Limited diffusion in poorly perfused regions	(62, 63)
	100–200 nm	Clathrin-mediated endocytosis, EPR retention	Enhanced tumor retention, reduced penetration	Liposomes, polymeric NPs	Liver and spleen accumulation	(48–52)
	> 500 nm	Macropinocytosis	Internalization independent of size limits	Micron-scale carriers	Poor penetration, clearance by MPS	(64, 65)
Shape	Spherical	Symmetric flow dynamics	Efficient internalization, lower margination	Spherical AuNPs, liposomes	Reduced endothelial adhesion	(64, 66)
	Rod-like (AR ~3-4)	Enhanced membrane wrapping, hydrodynamic lift	Increased uptake and circulation time	Au nanorods, rod-like MSNs	Orientation-dependent uptake	(66–68, 79, 80)
	Discoidal/ellipsoidal	Vascular margination, endothelial adhesion	Preferential tumor vascular localization	iNPG, discoidal silicon vectors	Limited deep tissue penetration	(53–57, 81)
Integrated Size-Shape	Injectable nanoparticle generator	Sequential transport across barriers	Decouples circulation, penetration, and uptake	Mesoporous silicon nanoparticles	Increased design and CMC complexity	(57, 82)
TOP Framework	Coordinated size + shape tuning	Exploitation of transport phenotypes	Tumor-selective accumulation beyond molecular targeting	Multistage silicon systems	Requires phenotype-specific optimization	(10, 43, 83)

**Figure 2:**
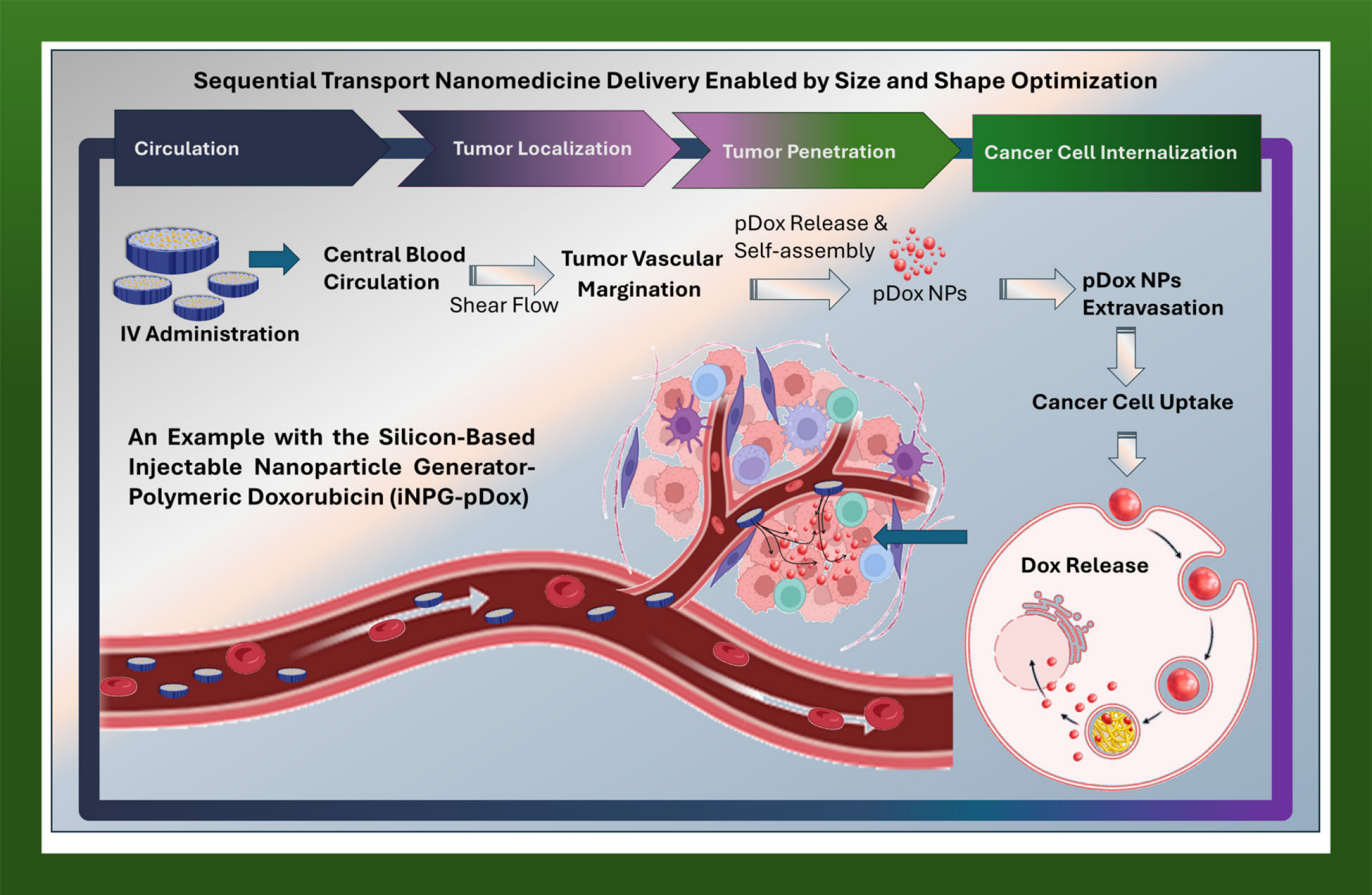
Sequential transport enabled by size and shape optimization overcoming multiple biological barriers to cancer drug delivery. Physicochemical properties of NBM, such as size and shape, are tuned to optimizing drug delivery based on dominant sequential transport barriers. The injectable nanoparticle generator iNPG-pDox system is used as a model example of this framework.

Beyond geometric considerations, the size and shape of nanoparticles are not just design parameters, but also critical physical variables that can be engineered to exploit the specific transport barriers and hemodynamic variations that differentiate cancer from healthy tissues. The shift from purely biochemical interactions to the physics of mass transport provides the framework of TOP that conceptualizes cancer as an order of physical and biological transport barriers and not solely as a genetic or molecular disease. In the TOP framework, the design of nanomedicines is a biomechanical problem, in which engineered NBM physically exploit the transport phenotypes in cancer tissues, optimizing circulation, transportation and therapeutic efficacy (10, 43, 83). In the TOP theory, delivery failure arises from barriers physical such as abnormal tumor vasculature, elevated interstitial pressure, heterogeneous ECM architecture, and biological such as the mononuclear phagocyte system endosomal sequestration, and drug efflux. These barriers act rather sequentially in the meaning that optimizing a single parameter independently such as NBM size or shape is insufficient for balancing the following transport limitations. TOP further emphasizes the distinction between systemic pharmacokinetics and tumor micro-pharmacokinetics. In this meaning, tumors with similar circulation profiles may exhibit profoundly different intratumoral drug distributions due to differences in vascular permeability, basement membrane composition, and ECM density (10). Importantly, TOP framework introduces the concept of transport phenotyping, whereby tumors are classified according to their dominant physical transport barriers rather than purely biological markers. This provides a rational basis for tailoring NBM design to tumor-specific delivery restrictions. Within this context, TOP argues that tumors can be classified by their unique transport barriers, naming their transport phenotype. Thus, the TOP framework is intrinsically linked with the cancer phenotype targeting concept aiming to design NBM that navigate and exploit specific physical transport barriers. Within this concept, size and shape are repositioned as critical parameters to coordinate drug delivery with release kinetics and overcome transport barriers encountered in solid tumors. An example of sequential transport phenotype targeting design is the aforementioned iNPG-pDox system (**Figure 2**). This design strategy reduced systemic toxicity and bypassed drug efflux pumps that drive multidrug resistance, leading to highly effective tumor cell death and functional cures in animal models of metastatic breast cancer (42). Beyond the iNPG-pDox strategy, various MSVs are designed to respond to tumor-specific stimuli (84). MSVs typically employ TME stimuli-responsive transformations in NBM size, surface chemistry, or structural integrity. Such changes enable stepwise navigation through tumor vasculature, extracellular matrix, and intracellular compartments. Multistage systems, such as biodegradable multifunctional mesoporous silica nanoparticles have been reported in which long-circulating, carbon dots-loaded MSNs first accumulate within tumors. Subsequently, these MSNs undergo pH-triggered disassembly to release carbon dots-based therapeutic complexes, followed by cellular targeting via a conjugated peptide and then the cytosolic transport of doxorubicin (85). Such TME-responsive platforms demonstrate improved tumor penetration and therapeutic efficacy in preclinical models by enabling stepwise navigation through vascular, extracellular, and intracellular barriers. In another strategy, gelatin nanoparticles (around 100 nm) carrying quantum dots (QDs around 10 nm) undergo protease-mediated degradation within the TME to release the QDs. Owing to small size, these QD particles diffuse more efficiently through the dense tumor interstitial matrix and achieve deeper tumor penetration (82). These studies highlight that sequential, stimulus-responsive NBM are promising systems, aligning with the TOP framework and requiring sequential optimization of physicochemical properties to navigate multiple transport barriers.

Rational NBM design therefore requires the joint optimization of size and shape to enhance tumor-selective delivery while limiting off-target accumulation and systemic toxicity, particularly for small molecules, peptide, and protein payloads. Importantly, optimal size and shape combinations are tumor dependent. Highly vascularized and permeable tumors, such as hepatocellular carcinoma and non-small cell lung cancer, favor small, rapidly diffusing nanoparticles in the 10–100 nm range (81, 86–88), whereas desmoplastic tumors like pancreatic ductal adenocarcinoma may benefit from larger NBM architectures capable of interacting with dense stromal environments or modulating ECM barriers (89, 90). Tumors with poor lymphatic drainage, including glioblastoma and triple-negative breast cancer, can further exploit prolonged intratumoral retention of appropriately engineered carriers (91, 92). While size and shape dictate how NBM navigate physical transport barriers, surface chemistry ultimately governs their biological fate, including circulation stability, immune recognition, and cell-specific uptake. The following section therefore focuses on chemical functionalization strategies that impart biocompatibility, targeting specificity, and responsiveness to the tumor microenvironment. Overall, NBM size and shape act as main determinants of transport behavior across multiple biological barriers. These parameters influence the circulation half-life, vascular extravasation, interstitial penetration, and cellular uptake in a context-dependent manner. Within the TOP framework, size and geometry therefore represent foundational design variables that should be considered in relation to the structural and physiological characteristics of the tumor microenvironment.

### 2.2 Surface chemistry and functionalization as modulators of biological interactions

Among the most dynamic determinants of NBM performance is surface chemistry, which critically manages their biological identity and interactions following systemic administration. Unlike conventional small molecule drugs, NBM are rapidly redefined upon exposure to physiological environments through the adsorption of endogenous biomolecules, predominantly plasma proteins, resulting in the formation of a protein corona (93). This acquired biological interface critically influences immune recognition, circulation time, biodistribution, cellular uptake, and interaction with tumor versus healthy tissues (94). Notably, the composition and evolution of protein corona depend strongly on surface chemistry of NBM, as well as physiological context and disease state, making surface engineering a determinant of therapeutic fate (95). The protein corona is a dynamic structure comprising a tightly bound hard corona and a more rapidly exchanging soft corona. While the hard corona largely affects long-term biodistribution and immune interactions, the soft corona governs early cellular engagement and targeting efficiency (96). The type of protein corona shapes the behavior of NBM and has significant impact on their biological interactions. Depending on their surface properties, NBM may preferentially bind opsonins, such as immunoglobulins and complement proteins, resulting in rapid immune clearance by the mononuclear phagocyte system (97). Conversely, NBM coated with dysopsonins, such as albumin or apolipoproteins can promote immune evasion, extended circulation time, and even facilitate endothelial transcytosis or tumor homing (98). Thus, protein corona functions as a biological gatekeeper bridging the physiochemical design of NBM with biological interactions such as circulation, endothelial transportation, immune responses, and tumor accumulation.

The protein corona has critical implications for tumor-targeted NBM, by either enhancing or hindering their therapeutic effectiveness. Coronas’ composition can influence extravasation, penetration, internalization, and most importantly, the effectiveness of targeting ligands for receptor-specific binding to cancer cells. Surface functionalization strategies directly modulate corona composition and its downstream effects on targeting. Targeting ligands, including peptides, antibodies, and small molecules, may be sterically masked or conformationally altered by corona formation, reducing binding affinity and specificity (99, 100). For example, monoclonal antibody-functionalized polymeric nanocapsules exhibited compromised targeting of the humanized A33 monoclonal antibody (huA33 mAb) when dense human serum protein adsorption masked receptor accessibility (101). Conversely, ligand architecture can be exploited to overcome corona interference. Hyaluronic-acid functionalized metal-phenolic capsules demonstrated enhanced targeting of CD44-overexpressing MDA-MB-231 breast cancer cells. The extended polysaccharide chains of high molecular weight protruded beyond the corona and retained CD44 receptor engagement. In this case, corona rather than blocking hyaluronan affinity enhanced targeting to cancer cells (102). Understanding and controlling the complex structure-to-function interplay between nanoparticles, protein coronas, and targeting ligands in biological systems is essential for improving targeting strategies of sensitive payloads such as small molecules, proteins and peptides. These observations highlight that effective targeting and uptake depend not only on ligand identity but also on ligand length, density, and spatial presentation relative to the corona.

**Figure 3:**
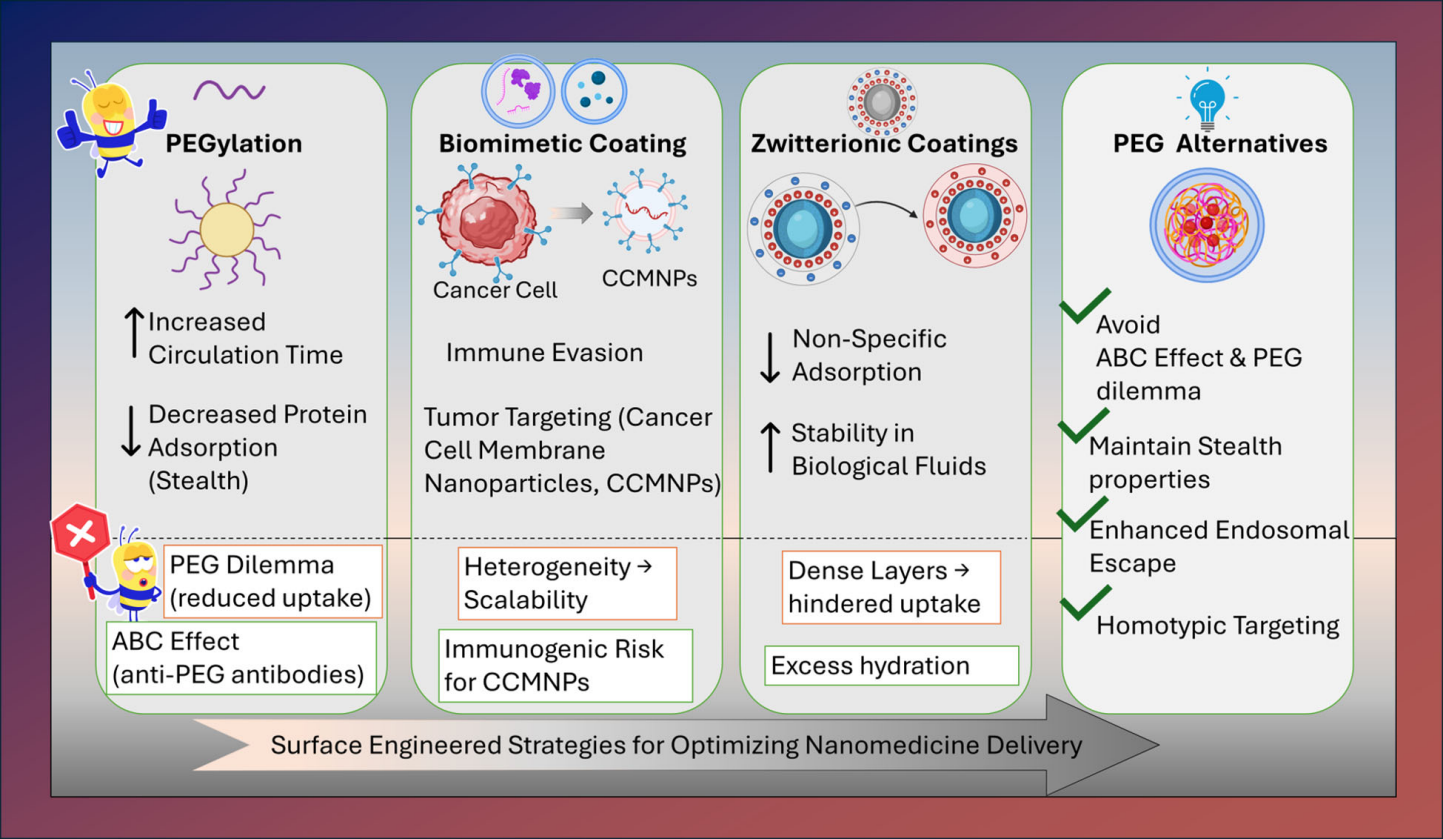
Surface chemistry and functionalization strategies for modulating biological interactions of NBM. The figure illustrates how surface chemistry governs stealth properties, ligand accessibility, and endosomal escape, with emphasis on reduced immune recognition and prolonged circulation.

Surface engineering strategies expand beyond PEGylation to cell membrane biomimetic and zwitterionic coatings to fine-tune corona composition, reduce immune recognition suppress opsonization, and prolong systemic circulation (summarized in **Figure 3**; **Table 3**) (106). As highlighted, the biological identity of NBM is continuously shaped by their interactions with endogenous proteins. PEGylation forms a dense, hydrophilic, and flexible layer that sterically hinders protein adsorption and opsonization, reducing recognition by the mononuclear phagocyte system. This “stealth” effect prolongs systemic circulation and limits nonspecific cellular uptake, enhancing the probability of passive tumor accumulation. Thus, surface chemistry plays a decisive role in enabling efficient delivery of small molecules and peptide/protein therapeutics. The clinical success of PEGylated liposomal doxorubicin (Doxil^®^) established PEGylation as a benchmark surface modification in oncology (125). NBM-based targeted formulations continue to illustrate the relevance of PEGylation strategies. In a PEGylated liposomal metformin system for hepatocellular carcinoma treatment, a high encapsulation efficiency (> 90%) was demonstrated with a mean hydrodynamic diameter of ~177 nm, and enhanced anticancer activity was achieved relative to the free drug, highlighting how PEGylation can simultaneously influence pharmacokinetic behavior and size control in tumor-targeted delivery (126). However, excessive PEG shielding can impede cellular uptake and ligand-receptor interactions, giving rise to the well-recognized “PEG dilemma”. The dense PEG brushes improve stealth properties but provide insufficient prevention from macrophage recognition, while the long PEG chains can increase therapeutic efficacy but hinder cellular uptake. This issue limits targeted drug delivery especially in solid tumors due to the abnormal TME with the gradient effect of acidic pH, temperature, and enzymes. PEG chain length, molecular weight, and grafting density must therefore be carefully optimized, and tumor-responsive dePEGylation strategies have emerged to preserve stealth during circulation while restoring uptake at the tumor site (103). Rational surface engineering can overcome these challenges especially for small-molecule delivery. In pH-responsive gold-polymer nanohybrid vesicular systems, surface functionalization enabled controlled paclitaxel release under acidic tumor conditions, enhancing intracellular drug availability while maintaining colloidal stability (104). Similarly, folic-acid functionalized gold nanorods achieved receptor-mediated uptake in folate-receptor overexpressing breast cancer cells, demonstrating how ligand presentation on anisotropic surfaces enhances selective internalization of chemotherapeutics (105). Beyond PEGylation, magnetic and stimulus-responsive surface chemistries further expand therapeutic functionality. Canagliflozin-loaded magnetic nanoparticles exploited both surface functionalization and external magnetic guidance to localize glucose inhibiting molecules within hypoxic tumor regions, significantly enhancing radiotherapy efficacy (40). Likewise, folic-acid-functionalized condensed magnetic nanoparticles enabled targeted doxorubicin delivery to folate-receptor-overexpressing tumor cells, improving intracellular accumulation while limiting off-target toxicity (121).

**Table 3:** Surface chemistry and functionalization strategies governing biological identity and tumor targeting of NBM.

Strategy category	Surface modification/mechanism	Representative NBM platforms	Key biological effects/design outcomes	Refs
Protein Corona Modulation	Hard/soft corona regulation via surface chemistry	Polymeric NPs, liposomes, MSNs	Controls immune recognition, circulation time, endothelial transport, and targeting efficiency	(93–99)
Stealth Coatings	PEGylation (optimized MW & density)	Liposomes (Doxil^®^), PLGA, Au NPs	Reduced opsonization, prolonged circulation, excessive PEG limits uptake (“PEG dilemma”)	(103, 104)
Tumor-Responsive Stealth	pH-/enzyme-cleavable PEG layers	Gold-polymer vehicles, smart NPs	Preserves circulation stealth, restores uptake in acidic TME	(104, 105)
Ligand Functionalization	Peptides (RGD, iRGD), antibodies, folate	PLGA NPs, Au NRs, liposomes	Receptor-mediated uptake, effectiveness depends on ligand density and corona exposure	(100–102, 105–108)
Zwitterionic Coatings	Phosphorylcholine, sulfobetaine, carboxybetaine	Polymeric NPs, lipid NPs	Ultra-low protein adsorption, dysopsonin-rich corona, preserved ligand accessibility	(109–112)
Biomimetic Surfaces	Cell membrane coating (RBC, platelet, cancer cell)	CMCNs, EV/exosome-based systems	Immune evasion, homotypic targeting, vascular adhesion, prolonged circulation	(113–117)
Protein-Based Nanocarriers	Albumin, ferritin, viral capsids	Abraxane^®^, ferritin NPs	Intrinsic biocompatibility, preserved receptor binding, protein payload protection	(118–120)
Magnetic/Stimulus-Responsive Surfaces	Magnetic targeting, external field guidance	Magnetic NPs	Spatial control, hypoxia targeting, radiosensitization	(40, 121)
Immunogenicity & ABC Mitigation	Alternative stealth, excipient-aware design	PEG alternatives, hybrid coatings	Avoids anti-PEG antibodies, mitigates accelerated blood clearance	(109–113, 120, 122–124)

Surface-engineered NBMss have been also extensively explored for the delivery of therapeutic proteins and peptides, where protection from enzymatic degradation and controlled binding affinity to target receptors are critical. This type of surface chemistry has been the basis for the development of Abraxane^®^ (nab-paclitaxel) that while still a small molecule, is a protein-based carrier system (118). The conjugation of peptide ligands, such as cyclic RGD, and cell-penetrating peptides to NBM has been widely explored to enhance tumor targeting, cellular uptake and therapeutic efficacy, including pH- and redox-responsive systems for drug delivery. For example, nanoparticles functionalized with cyclic RGD peptides improve recognition of αvβ3 integrin-overexpressing tumors, enabling enhanced delivery of chemotherapeutics and imaging agents (107). Tumor-penetrating peptides such as iRGD have been used to improve deep tumor penetration of nanocarriers by activating transvascular transport pathways and demonstrating translational relevance in solid tumor targeting (108). Protein NBM platforms, such as engineered ferritin, viral capsids, and albumin nanocarriers also serve as biocompatible carriers with inherent targeting and antigen delivery potential. Such NBM have emerged as particularly important targeting molecules, as their conformational flexibility can preserve receptor binding even in the presence of a protein corona (119). NBM have enabled systemic delivery of fragile protein therapeutics by shielding them from proteolysis while preserving bioactivity. Engineered nanocarriers have been developed for cytosolic delivery of therapeutic proteins, using rational surface modifications that protect proteins in circulation and promote release within the TME (127). On this basis, recent studies also highlight the role of liposomal surface architecture in influencing downstream biological responses. In a liposomal system developed for the co-delivery of β-carotene and doxorubicin against colorectal cancer, the β-carotene was integrated with lipid membrane and doxorubicin was encapsulated. The system demonstrated high drug encapsulation efficiency together with enhanced apoptosis induction and pronounced G0/G1 cell-cycle arrest in tumor cells (128).

PEGylation, despite its significant advantages, is associated with the accelerated blood clearance (ABC) phenomenon emerging from repeated administration, inducing anti-PEG antibodies, and rapid nanoparticle elimination by macrophages. The ABC effect results in reducing therapeutic efficacy and exacerbating systemic toxicity, by nanoparticles being rerouted to the liver and spleen (120). This limitation underscores the need for alternative stealth strategies, particularly for nanomedicines requiring repeated dosing. In pH-responsive tumor-adaptive nanoplatforms, surface functionalization with amphiphilic polymers enabled the morphological transformation of the NBM within the acidic TME. This formulation mitigated ABC-related effects by reducing repeated dosing and acting as a local reservoir for prolonged intratumor localization and release of chemotherapeutics (122). Beyond PEG, excipient-induced ABC effect can significantly implicate nanodrug delivery. Non-ionic surfactants such as poloxamer 188 (F68) and poloxamer 407 (F127) can systematically amplify anti-PEG immune responses when administered in combination with PEGylated liposomes and PEGylated liposomal doxorubicin (PLD). Pre-treatment with F68 amplified rapid nanoparticle clearance and reduced antitumor efficacy by over one-third, whereas F127 had minimal effect (123). This study identified excipient-specific differences in ABC effect signifying the possibility of inducing implications to cancer patients during chemotherapy cycles from the supportive formulation, such as antiemetics, parenteral nutrition, or adjuvants containing PEG derivatives. PEGylation is frequently employed to enhance systemic stability of protein or peptide drugs, but its effects on ligand binding affinity, transfection efficiency, and tumor uptake must be carefully considered.

To avoid PEG-associated immunogenicity, zwitterionic coatings, based on phosphorylcholine, sulfobetaine, or carboxybetaine chemistries, offer comparable resistance to nonspecific protein adsorption, improving stealth properties (124). Zwitterionic molecules occupy both positive and negative surface charges within the same surface, creating a strong and stable hydration layer around the NBM (109). Thus, such coatings limit electrostatic and hydrophobic interactions that drive nonspecific protein adsorption (110, 111). These coatings favor dysopsonin-rich coronas, mainly serum albumin and certain apolipoproteins, preserve ligand accessibility, and support receptor-mediated uptake. Thus, they offer a critical baseline for downstream functionalization such as peptide, aptamer, proteins, and small molecules for optimal tumor delivery mechanisms (112). Biomimetic surfaces further extend this concept by coating NBM with natural cell membranes derived from erythrocytes, platelets, leukocytes, or cancer cells, offering advantages in homotypic targeting. Such coatings confer immune evasion, prolonged circulation, and tissue-specific tropism through inherited membrane proteins, including CD47-mediated “self” signaling and homotypic tumor recognition (113). Such effects influence biological interactions between cells and NBM that inherit the same “parental” properties. For example, CD47-mebrane rich NBM can present increased circulation time and immune evasion, while cancer cell membrane-coated NBM can facilitate tumor targeting (114–116). Cell membrane-coated nanoparticles (CMCNs) have demonstrated enhanced delivery of small-molecule and peptide therapeutics by exploiting inherited properties by their parental membranes such as vascular adhesion (platelet-membranes), inflammatory homing (leukocytes-membranes), or tumor-specific interactions (stem cells-membranes) that are difficult to replicate with synthetic ligand chemistry. Additionally, exosome/EV-based CMCNs are considered the most promising in delivering small molecules to tumors, exploiting the intrinsic tropism from the parental cells (116, 117). Exosomes deriving from HT1080 and HeLa cancer cells for Doxil^®^ delivery (Doxil^®^-Exosomes, D-exo), presented increased homotypic targeting in HT1080-bearing nude mice enhancing Doxil^®^ therapeutic efficacy and suppressing tumor growth. The D-exo were characterized by proteomic analysis on the integrin expression patterns, revealing the effect of distinct integrins in mediating selective fusion of exosomes (117).

Nevertheless, biomimetic and exosome-based systems introduce translational challenges, including batch-to-batch heterogeneity, complex cargo composition, immunogenic risks from tumor-derived components, and scalability constraints under GMP manufacturing (129, 130). Emerging hybrid strategies that combine synthetic surface chemistries with selected cell membrane coating functionalities may offer a balance between biological performance and clinical reproducibility (131). In summary, surface chemistry serves as the principal interface between NBM and biological systems. Rational surface engineering, integrated with size and shape optimization, enables nanomedicines to navigate complex TMEs, enhance delivery of small molecules and peptide/protein drugs, and move closer to clinically translatable cancer therapies. Together, these examples illustrate the role of surface chemistry in governing the biological identity of NBM after systemic administration. Interactions with plasma proteins, immune components, and tumor-associated biomolecules dynamically reshape nanoparticle behavior *in vivo*. Within the TOP framework, surface functionalization therefore serves as a critical interface parameter that modulates circulation stability, immune evasion, and tumor-specific interactions, ultimately influencing the efficiency of transport across sequential transport barriers.

## 3 Translational bottlenecks, solutions and future perspectives

### 3.1 Translational bottlenecks and potential solutions

Beyond conceptual advances in NBM design, several biological and technological challenges persist and should be addressed to enable successful clinical translation. First, tumor heterogeneity remains a major limitation affecting vascular permeability, stromal density, immune infiltration, and interstitial pressure. Such variations can significantly alter NBM transport behaviour across different tumor types and even within individual lesions. Such variabilities significantly limit the predictive value of conventional NBM design strategies based primarily on the EPR effect. Addressing tumor heterogeneity will likely require the development of adaptive or phenotype-guided NBM platforms capable of responding to dynamic TME conditions and transport barriers. Second, recent analyses of NBM-based cancer therapeutics have emphasized that innovative material design alone is not sufficient to ensure successful clinical translation, underscoring the importance of scalable and regulatorily complaint formulation and manufacturing strategies across diverse NBM classes are also needed (3, 129, 130, 132). For example, the sequential transport NBM represent a promising strategy, with enhanced intratumoral drug delivery demonstrated in preclinical models, yet only a few candidates have proceeded to clinical stages (e.g., ML-016) (132). This underscores the need to develop NBMs as drug products, taking into account formulation scale-up, manufacturing, and regulatory considerations, rather than viewing them solely as nanomaterials. In this regard, formulation composition and process design are as critical as the structural design of NBMs. Early engagement with regulatory agencies (e.g., a pre-IND meeting with the FDA) and consultation with a contract manufacturing organization (CMO) can help de-risk the development process and ensure that translational pathways remain aligned from an early stage.

Furthermore, sophisticated and human-relevant preclinical evaluation remains lacking for accurate prediction of biodistribution, clearance pathways, and toxicity profiles. Although 2D *in vitro* models remain essential for early-stage screening due to their simplicity and high-throughput capability, their ability to predict NBM behavior in more complex biological environments is very limited. Advanced experimental platforms, including tumor-on-chip microfluidic systems, 3D organotypic tumor cultures, and patient-derived tumor explants, have therefore been developed to better replicate the dynamic TME and provide a valuable alternative between 2D *in vitro* assays and *in vivo* tumor models. Nevertheless, *in vivo* animal models remain a critical component for the evaluation of systemic biodistribution, immune interactions, and tumor penetration dynamics (132). The integration of *in vivo* models with quantitative imaging approaches, pharmacokinetic modelling, and computational methods may generate more predictive data models. Such integrative approaches may narrow the translational gap between mechanistic transport insights and the development of clinically effective nanomedicine.

### 3.2 Future perspectives

**Figure 4:**
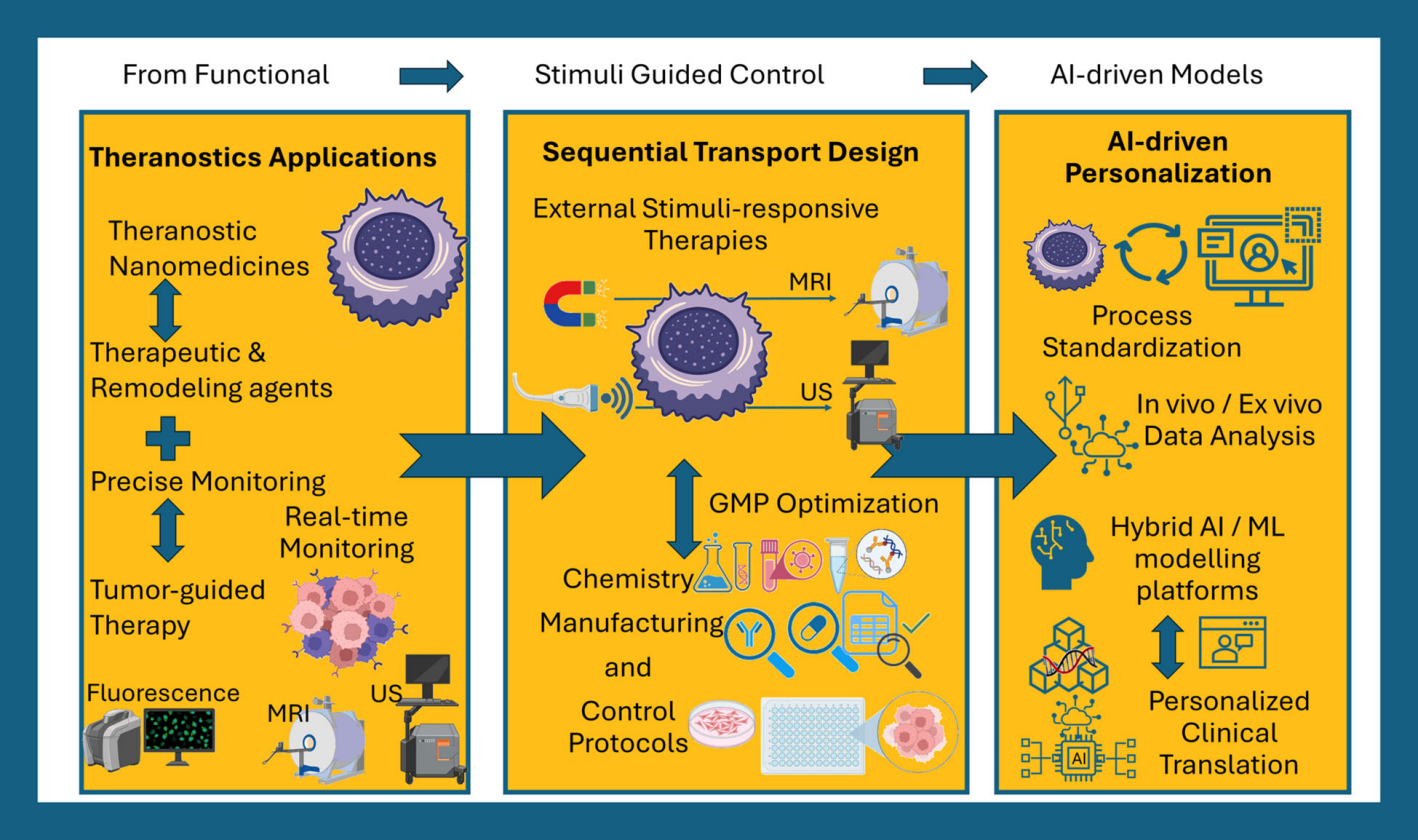
Future directions in structure-to-function NBM. The evolution of NBM design is presented through sequential transport and theranostics that integrate physicochemical optimization with external stimuli and real-time monitoring. AI-guided feedback may enable adaptive modulation of nanomedicine behavior in response to tumor transport phenotypes. Such design strategies could support personalized and dynamically evolving delivery systems that move beyond static NBM design principles.

NBM can be effectively designed to remodel the TME of various cancer types by tailoring their physicochemical properties to navigate TME barriers in combination with small molecules and peptide/protein targeting. The direct connection between the NBM structure and their biological function can be exploited for modulating TME properties to enhance drug penetration and immune responses. The development of advanced NBMs capable of integrating therapeutic and diagnostic functionalities represents a key direction for improving clinical translation and enabling more personalized treatment strategies (3, 129, 130). A conceptual roadmap illustrating future directions in multifunctional and data-driven NBM design is presented in **Figure 4**. Such NBM allows the monitoring of the tumor’s response to treatment allowing real-time adaptation of therapeutic strategies. On the structure-to-function axis, the most important shift will be toward multifunctional theranostics that couple precise tumor targeting systems with real-time diagnostic monitoring. Theranostic NBM presents ideal candidates for peptide/protein molecule delivery since apart from protection and selectivity they offer monitored localization and cytosolic release which enables real-time guided delivery. Such feedback may allow for rapid adaptation, either of the dose (e.g., reduce dose levels) or of the nanotherapeutic, including small molecules, and peptide/protein-based therapeutics, throughout the treatment course.

The combinations of theranostic NBM with externally applied stimuli such as magnetic fields, focused ultrasound, and NIR light provide an extra level of control and strengthen theranostic actions. Magnetic nanoparticles can be used concurrently for MRI contrast, magnetically guided accumulation, and thermal stimulation to promote local uptake or enhance endosomal escape. Focused ultrasound and sonoporation offer clinically available, non-invasive methods to rapidly increase vascular permeability and activate release from microbubble-coupled or ultrasound-responsive theranostic nanoparticles (133). Additionally, ultrasound imaging can integrate small molecule delivery with nanoparticle monitoring. The NIR light activation of photothermal and photodynamic theranostic nanoparticles such as gold NPs continues to advance with such therapies being able to induce immune activation that promotes cancer cell death (134). The real opportunity in protein theranostic nanoparticles lies in the integration of monitoring and control of therapeutics under the effect of external stimuli in a spatially and temporally tuned manner. This way, NBM imaging confirm particle accumulation while an external trigger converts the carrier from stealth to active state (e.g., via cleavable PEG or shape-changing structures). Thus, small molecules or protein payloads are released in a focused, high-efficacy window. This synergistic effect can greatly assist in addressing crucial TME challenges simultaneously such as heterogeneous intratumoral delivery and systemic toxicity. The combinational design of theranostic NBM and local physicochemical stimuli can increase modulation of TME and target functional tumor phenotypes such as transport phenomena.

AI-modelling represents a distinct direction for NBM and an essential tool for future research. Machine learning (ML) and deep learning can influence the design of NBM by providing predictions of protein corona evolution, optimized structures such as size and shape for long circulation, tumor penetration probability of the NBM, and release kinetics. The synergy between experimental science and AI modeling should be critically promoted in the future for the models to operate in a physicochemically and biologically informed manner. Such hybrid approaches enable the documentation and analysis of important structure-to-function responses in AI models such as transport phenomena, fluid dynamics for margination and extravasation, and diffusion of nanoparticles for interstitial transport (135). The computational translation of research data will enable the creation of data-driven models for generalized insights in the connection of structure-to-function with targeting TME of varying tumor types. Hybrid AI-models can also be fed with *in vitro* and ex vivo data generated from animal studies and patient-derived tissues. Such models would allow the accumulation and analysis of a great number of parameters from various NBM to provide intelligent guidance and limit unnecessary animal testing. Critical advances will come through technology platforms that make structure-to-function rules accessible to interdisciplinary teams. In this regard, open databases of nanoparticle physicochemical properties, protein corona characteristics across various animal species and strains and disease states, and linked release kinetic outcomes should be developed. Such models would facilitate scientists on the rapid development of optimized nanomedicines and clinicians for reliable predictions on their dose and efficacy. Patient-specific AI-models that combine imaging, metabolomics, and therapeutic outcomes could enable in silico testing of nanomedicines and dosing regimens prior to administration, thereby reducing risks and improving clinical trial efficiency. However, training of these models introduces challenges related to standardization and ethics, which depend heavily on data selection criteria. If issues surrounding data quality, infrastructure access, and cost are not carefully managed, such approaches could inadvertently exacerbate healthcare inequalities.

## 4 Conclusion

The physicochemical design of NBM fundamentally determines their biological performance and therapeutic outcome in solid tumors. Across the studies discussed in this review, it is evident that no single physicochemical parameter can independently predict efficacy due to the pronounced biological and mechanical heterogeneity of TME. Thus, size, shape, and surface chemistry rather act as integrated active factors to regulate biological responses. These parameters regulate transport through biological barriers, cellular uptake, tumor penetration, and immune interactions, highlighting that the structure of NBM is inseparable from their function. A central conclusion of this review is that tumor-specific biological and transportational barriers necessitate tailored NBM designs. Generalized rules provide a framework, but meaningful therapeutic benefit requires nanomedicines engineered for the specific pathophysiology of TME. For example, size is related to transport pathways and biodistribution, surface charge affects interaction with cellular membranes and systemic clearance, surface ligand modification dominates the active transport mechanisms and TME targeting. Consequently, the performance of nanomedicines cannot be separated from NBM design. The application of the structure-to-function relationship into NBM was exploited to protect small molecules, peptide and protein molecules, enhance their stability, and enable targeted delivery across the diverse TMEs. While generalizable design rules provide a foundation, effective NBM performance requires exploiting transport phenomena and functional tumor phenotypes such as abnormal vascular dynamics, stromal barriers, and intracellular transportation constraints that govern therapeutic access within TME (136).

Despite significant advances in innovative NBM design, remaining challenges limit their clinical translation, those include but not limit to addressing tumor heterogeneity, drug product scale-up manufacturing, and predictive preclinical evaluations. Overcoming these barriers will require improved experimental validation, integrated preclinical models, and scalable formulation development strategies. Looking forward, adaptable and responsive NBM platforms would overcome the spatial heterogeneity and dynamic nature of solid tumors. Future progress may benefit from the integration of physicochemical design principles with tumor-specific engineering strategies that exploit transport phenotypes, biological vulnerabilities, and TME constraints. These advances may enable the development of next-generation nanomedicines capable of achieving more reliable and clinically effective cancer therapies.
